# Maximizing participation while preserving the normative order of the service: The autonomy and porosity of service encounters

**DOI:** 10.1177/14614456241292723

**Published:** 2025-02-02

**Authors:** Lorenza Mondada

**Affiliations:** University of Basel, Switzerland

**Keywords:** Conversation analysis, intersecting interactions, multiactivity, multiple participation frameworks, order of queuing, social interaction

## Abstract

While Conversation Analysis has mostly focused on single autonomous episodes of social interaction, characterized by their overall structural organization delimited by openings and closings, alternative forms of organization have also, although more rarely, been pinpointed, involving several interactions intersecting together. This paper explores the systematic practical and normative way in which participants orient to the autonomy of encounters while at the same time dealing with their porosity, that is, with emerging intersecting actions impinging on the current course of interaction. The empirical analysis focuses on service encounters at the market, in France, in which several customers assemble at the counter, waiting to be served. The paper analyzes how the salesperson manages the encounter with a current customer, while addressing new customers, by initiating intersecting sequences of actions. it also show what whereas some intersecting actions are treated as unproblematic, others are normatively oriented to as violating the order of the service. These observations enable us to analyze the autonomy versus porosity of the encounter as a members’ concern and study how they deal with these alternative organizations while maintaining a normative sense of the order of service.

## Introduction

The autonomy and integrity of single episodes of social interaction have been the focus of Conversation Analysis (CA) since its inception—emphasizing the importance of conversation’s overall structural organization with a clear beginning and ending achieved by opening and closing sequences. However, other forms of social organization reveal a certain porosity between interactions that impinge on each other, and that even intersect with each other. This paper is about one of these alternative forms of organization, as it is managed and oriented to practically and normatively in situ by the participants.

The empirical part of this paper focuses on service encounters, as a perspicuous setting for examining both the autonomy and the porosity of social interactions. In service encounters, the salesperson often serves a customer at a time, orienting to the integrity of the encounter as achieving the quality of the service. However, in specific circumstances, for example, when several customers assemble in front of the counter to be served, the salesperson might engage in managing not only one customer at a time, but also sequences of actions with customers queuing for their turn: the salesperson displays devoting specific attention to the current customer, while displaying some attention to the next customers—thus progressing the current transaction as well as addressing the queuing customers. In other words, the salesperson might display an orientation *both* to the autonomy and the priority of one encounter in particular, *and* to its porosity, maximizing the participation of other participants. While this might be implemented in different ways, in this paper, I focus on the methodic organization in which the salesperson orients to and actively preserves the priority and progression of one customer at a time, while punctually interacting with other customers.

This form of multi-participatory organization does not only respond to practical, professional, and economical issues; it also constitutes a way in which participants actively orient to the integrity and autonomy of social interaction, in a setting in which encounters present some porosities, hinting at possible alternative models of organization, in which several parties could be attended, without waiting one party to have completed the interaction. While CA has largely privileged the organizational autonomy of social interactions that begin and unfold until they are brought to closing, alternative forms of organization have been pointed at, although being less studied, such as diverse types of multiactivity, in which one or more participants engage in different activities in parallel or alternating ways. Describing a form of activity in which one participant manages both the integrity of the interaction with a participant and its local porosity with other participants, enables us to better understand alternative modes of temporal and sequential organization as well as their normative dimension. The latter is particularly observable in how participants orient to moments of porosity in the current encounter, treating it as legitimate or problematic. This produces analytical insights into the autonomy and integrity of the encounter as a members’ concern.

The paper deals with a collection of cases video-recorded at the counter at a Christmas market, in which a salesperson engaged in serving a customer addresses other possible customers queuing, maximizing their participation and involvement in the selling/buying activity. The collection includes different types of sequences initiated either by the seller or by the customer(s), revealing a variety of actions that can be considered by them—sometimes in diverging ways—as possible and legitimate; it also includes sequences that the seller and sometimes also the customers treat as problematic. In this way the analysis demonstrates how the integrity of the exchange is considered as preserved despite the seller addresses other recipients, and by contrast how some actions are normatively oriented as violating it.

## Background

This study focuses on an empirical setting—service encounters in which the salesperson faces organizational challenges in managing at the same time an ongoing transaction with a customer party and multiple other customers waiting and queuing—in order to reflect tono a general conceptual issue, regarding the sequential and normative organization of social interaction(s) as single and autonomous versus multiple and porous events. In the latter case, participants engaged in one course of interaction might be confronted with multiple other participants who might be punctually addressed or, more radically, with other interactions that intersect with the current one.

The general issue tackled by the analysis concerns the organizational and normative order of multiple partially concomitant social interactions. The CA literature deals mostly with single autonomous interactions, characterized by “the overall structural organization” characteristic “of the unit ‘a single conversation’” (Schegloff, 2002: 272, 2007: 263, Sacks, 1992: 157), which emerges from the opening sequences, achieving the coordinated entry of the participants in a joint activity (Schegloff, 1968, 1986) and is completed by the closing sequences (Schegloff and Sacks, 1973) that likewise organize the coordinated and aligned ending of the participants’ engagements. Thus, openings and closings delimit the temporal and praxeological frontiers of social interaction (Robinson, 2012). Most of the phenomena studied in CA are situated within these two moments.

By contrast, how single instances of social interactions are embedded in larger praxeological and interactional contexts, within the flow of social life, without any time out, is much less investigated. This includes what happens before the opening (Mondada, 2016), and after the closing (Mondada, 2008) of an interaction, how participants engage in multiple activities “at the same time,” as in multi-activity (Haddington et al., 2014), how an interaction can split in two or more and then recompose (as in schisms, Egbert, 1997), how participants in one interaction overhear another one and exploit it within their own activity (M.H. Goodwin, 1996; Hänggi, 2022; Mondada, 2021), or how one interaction can intersect with another one—as studied here. These phenomena show that beside the autonomy of single conversations, other forms of interaction exist, that address the continuous flow of social life and the multiple engagements of the interactants.

This paper deals with the organization of *intersecting interactions*, that is, contextual and praxeological configurations in which during an ongoing social interaction, other participants intervene, intersect, interject with it, in minimal or expanded ways. These configurations raise the problem of the autonomy and integrity of a single interaction as a local problem, achieved by and for the participants: How porous can be an ongoing interaction? Are there normative and practical orientations to the autonomy of a single interaction? What are the normative and practical treatments of multiple actions interjected in a current interaction? Which potentialities and constraints are revealed by local normative orientations of the participants?

Some of these questions have been addressed in terms of *multiactivity* (Haddington et al., 2014), although this notion covers very different forms of multiple concomitant activities, which do not all concern multiple participation frameworks and multiple interactions intersecting together. While most cases studied in terms of multiactivity concern a group of participants involved in more than one activity—typically one in which talk is predominant, and another that can be mostly carried out with manual or embodied actions (like chatting while driving or cooking)—the configuration addressed here concerns multiple interactions, involving different participation frameworks, that are locally punctually embedded one in another, while keeping distinct sequential trajectories. In the literature similar issues have been mentioned in relation to phone calls interjecting some face-to-face ongoing conversation—addressing how participants manage calls during a conversation (Relieu and Morel, 2004), incoming calls while driving (Haddington and Rauniomaa, 2011), outgoing calls during a service encounter (Ticca, 2014), or various types of exterior summons during a videocall (Licoppe and Tuncer, 2014). These are all cases in which a call, as an external event, intrudes in the ongoing conversation. By contrast, in the cases targeted in this paper, I am interested in two or more intersecting face-to-face interactions—where the new incoming participant(s) can be seen and heard by the current participants, and therefore their emergent participation is witnessed and somehow negotiated by all the parties. Some workplace settings are pervasively characterized by this kind of multiple interactions, such as the airport control room described by M.H. Goodwin (1996) in which professional skillfulness relies on the capacity of integrating various incoming announcements, notifications, and sources of information overhearable in the room. Another workplace configuration in which several interactions are intersected is constituted by ongoing professional activities such as surgical operations in which surgeons manage the task with their team, while at the same time being available for an external audience, who has to figure out when and how to legitimately insert questions and comments, thanks to the mediation of some expert (Lindwall and Lymer, 2014; Mondada, 2014, 2023a). In the cases studied here, there is no mediation, but here-and-now local engagements of the participants initiating—or blocking—extended participation. Thus, despite these existing studies, the question of how these multiple intersecting interactions are ordered, practically and normatively addressed and managed remains largely understudied.

The empirical situated activity considered in this paper, service encounters (Aston, 1988; Fox et al., 2023; Merritt, 1976), constitutes a perspicuous setting (Garfinkel, 2002) for observing the systematicity of these multiple intersecting orders. The analyses focus on a specific configuration will be the focus of the analyses, emerging when various customers assemble in front of the counter, waiting and queuing for being served, and are addressed by the salesperson in the course of an ongoing encounter. The configuration is enabled by what I call *encounters-in-a-series* (Mondada submitted), that is, an organization of the salespersons’ work characterized by the continuous unfolding of one encounter after the other, without any time out, in which the closing of the previous encounter is immediately followed by the opening of the next one. This seriality of the encounters generates several types of porosity between encounters, interesting for exploring how far the integrity and autonomy of a single episode of interaction is actually oriented to by the participants, and how far it can be opened intersecting and embedded other episodes or fragments of to other interactions. One type of porosity is observable between the closing of the previous and the opening of the next encounter, the latter often happening when the former is not yet completed (Mondada, in press). Another type of porosity, explored in this paper, consists in the fact that in the middle of an encounter, with a current customer, another customer is addressed, in more punctual or more extended sequences of talk. In the CA literature, the analysis of some service encounters has recognized similar phenomena, for example when two sellers talk together and suspend their conversation to welcome a customer, thereby orienting to the priority of the sales encounter (documented by two examples in Kamunen and Haddington, 2020, 90–93, 112–115), or when the restaurant owner at the till is talking with a customer and manages to exchange money with another customer (documented by one example in Raymond and Lerner, 2014, 231–235). However, these configurations are not the main or only focus of these previous studies, so that a systematic investigation of the sequential order of these phenomena is still missing.

## Data

The data come from a larger corpus of service encounters video-recorded in Europe. The study focuses on one stand at a Christmas market, in Alsace (France), selling high quality chocolate. The data have been recorded with the collaboration of the seller and the informed consent of the customers.

A specific organization of the queue and the ecology of the counter favors the target phenomenon. The counter is quite long, presenting an abundant diversity of chocolates. The customers queue along the counter (vs perpendicularly to it) in such a way that the waiting customers can look at the products. Consequently, the queue is not linear, and customers just progressively arrange at the counter—the order of the queue being informally reckoned by the seller. This enables the co-present parties to overhear and oversee what is going on in the current transaction—a feature that is used by the seller to utter product presentations and explications that can be heard by diverse parties. Contrary to other market stalls where customers might select and fetch products themselves, here the access to products depends on the unique seller’s service.

This ecology, which is frequent in markets and food stores, constitutes a perspicuous setting (Garfinkel, 2002) for the phenomenon of interest here: the seller engages *both* in serving one customer at a time, in what is recognizably designed as a single continuous main transaction, *and* in addressing other customers in parallel, within (sequences of) actions publicly witnessable as embedded in the ongoing transaction without possibly competing with it. The challenge for the seller is to organize the main transaction and the embedded ones in such a way that the integrity of the main transaction is preserved, while at the same time maximizing recipiency and participation of other parties.

The practical seller’s challenge, which can be formulated as dilemmas of service, has deeper consequences in terms of sequential organization. The seller preserves the successive order of the service, in which they recognize legitimate and entitled current interlocutors, but at the same time addresses multiple next interlocutors who will be served later in the order of the queue. Various encounters are thus intersected, although clearly hierarchized and sequentialized. The practical problem of the seller creates a complex sequential organization that is neither the linear order of a single interaction with one customer at a time, nor the simultaneous order of multiactivity. This paper focuses on the work of the seller as a perspicuous phenomenon to unpack these complex orders of sequentiality, showing both their organizational and normative aspects.

## Analysis

The analysis focuses on actions that are recurrently initiated by the seller addressing other customers than the current ones, and considers how they are accountably positioned and treated as legitimate, as respecting the rights of the current customer as well as the priority and autonomy of the ongoing transaction. The analysis also considers the way these sequences of actions are responsive to and responded to by different parties and how they can expand in ways that are normatively treated as problematic by the seller and the queuing participants. In this way, these actions reveal the participants’ orientations to the order of the queue, the order of encounters-in-a-series, as well as the integrity of single encounters.

Some actions deal with the management of co-presence and waiting (§4.1), such as greetings (§4.1.1) and announcements of imminent availability (§4.1.2). Other actions engage in the management of service (§4.2), such as offers to taste a sample of a product (§4.2.1) and their expansions (§4.2.2). While greetings are never considered as problematic by the current customer, expansions following the acceptance of offers to taste are sometimes treated as normatively problematic. This distinctive treatments of embedded sequences of actions in the current encounter thus reveal the fine-tuned normative orientations of the participants in policing the integrity of the encounter.

### Managing co-presence and waiting

#### Greetings

A simple way to address new customers approaching the counter is to initiate a greeting sequence, embedded within the current transaction. These greetings are sensitive to the trajectory of the new customer(s) approaching as well as to the sequential unfolding of the ongoing encounter.

We join a first instance: a customer (CUS) joins the stand (1) and the seller initiates the encounter with some greetings (2), reciprocated (3). As the transaction continues, with an offer to taste (4), two new customers (NCU1, NCU2) approach the counter and are greeted (10), before the seller continues with the initial customer. In the transcripts, the gray zones indicate the intersected actions.

**(1)** (**48.24**-**49.52—CLI9-CLI10.1/2)**

**Figure:**
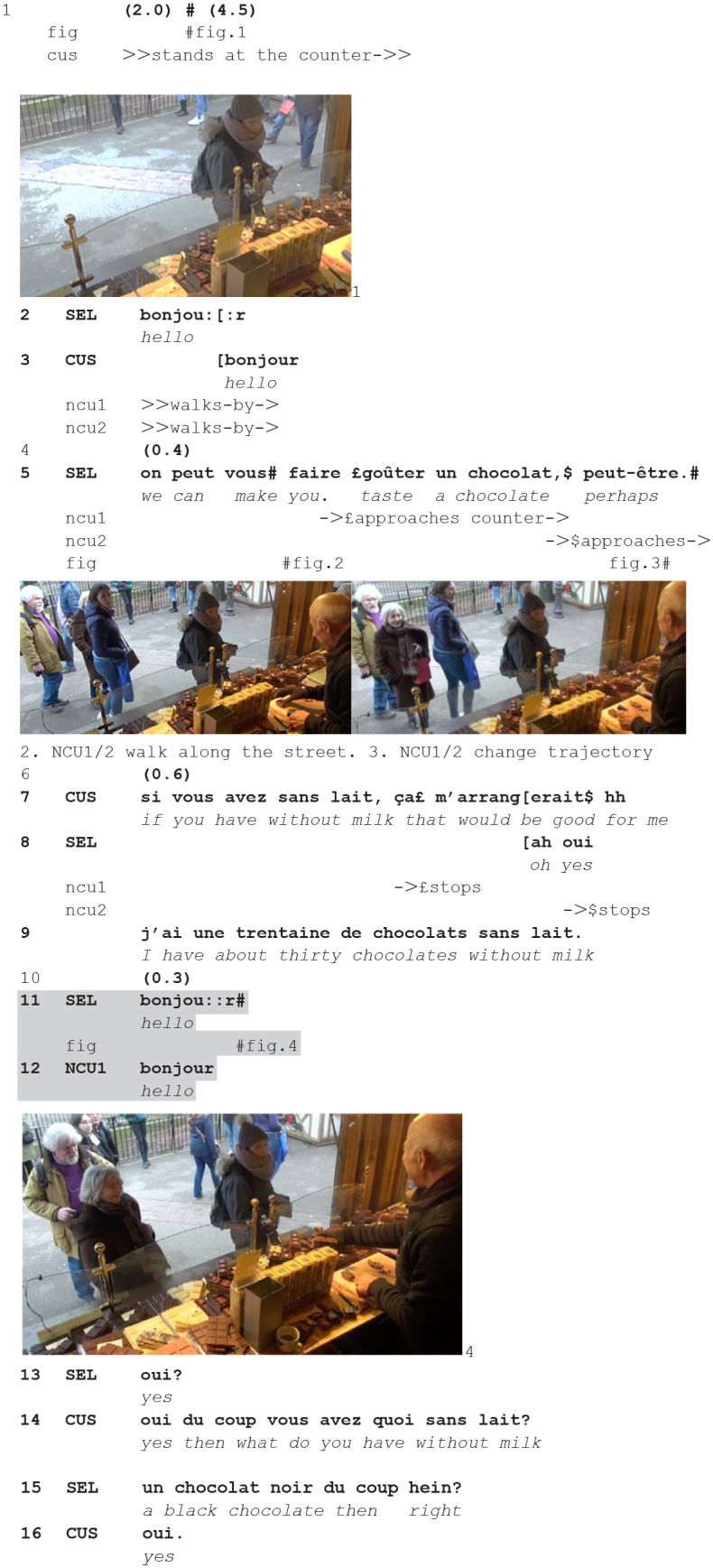


The customer (CUS) is standing for some time at the counter (1, fig. 1), waiting for the seller to turn to her. When he does so, greeting (2) and then offering a taste (3), other persons are passing by the stall along the road (fig. 2). While one passer-by continues her way forward, two other (NCU1, NCU2) pivot, changing trajectory and perpendicularly stepping toward the counter (5–7, fig. 3), finally stopping in front of it (7–8).

The seller sees the two persons approaching, and stopping at the counter—now becoming new/future/possible customers. As they stop the current customer is uttering a request (7). The seller skillfully places his greeting *after* he responds to the customer (9), thereby creating a next sequential slot in which she can expand her request. The customer does not immediately respond (10) and the seller uses that sequential environment to greet the new customers (11), reciprocated by one of them (12). Thus, he places the greeting at a sequential point in which he is not expected to produce any action, the next turn being expected by the customer (fig. 4).

As soon as the greeting sequence is completed, the seller explicitly addresses the customer (13): his interrogative *oui?*/“yes” is both orienting to the response the customer has not yet given and making observable that he is paying attention back to her. She responds, and her turn-initial *oui*/“yes” might register both aspects. They continue in elaborating on the choice of the black chocolate she wants.

Likewise, in the next fragment, the seller addresses a new customer at the first possible sequential occasion—here in a different sequential environment, the payment.

**(2**) (**56.34**-**56.55—Cli10.1/2-Cli12)**

**Figure:**
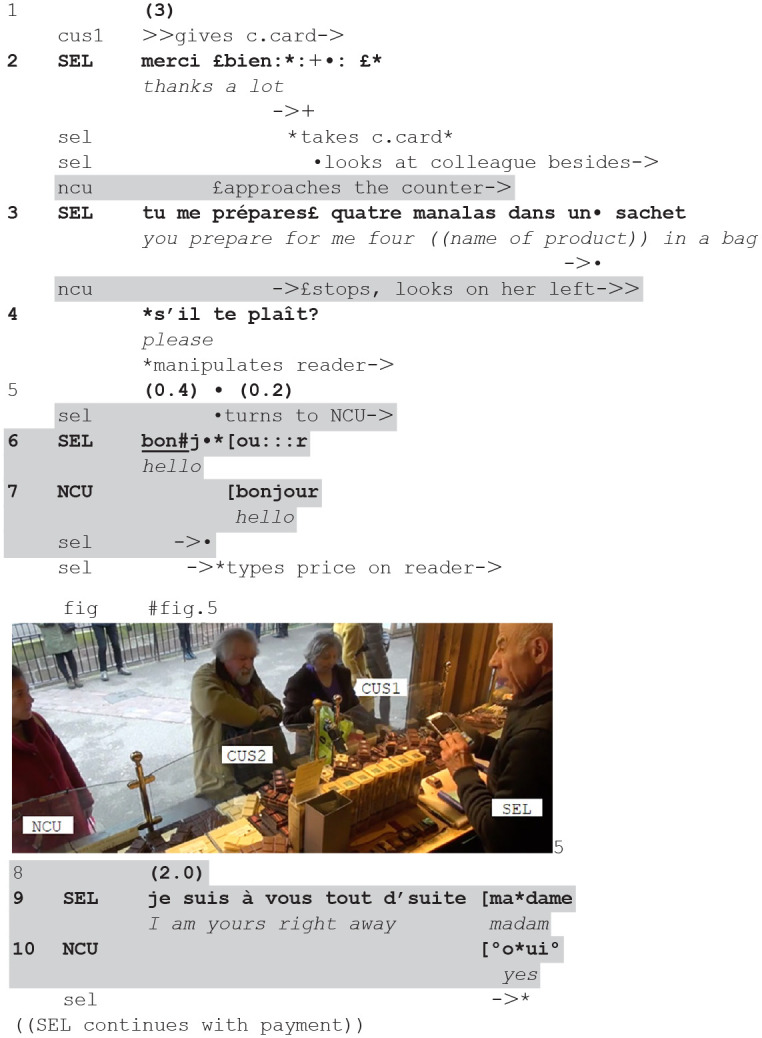


The current customers are paying their purchase. The seller takes their credit card (2) and while manipulating the card reader, he asks his colleague to prepare the bag with the products (3). At this point, a passer-by approaches (2) and stops (3), becoming a new possible customer. She looks at the counter/the other customers (see fig. 5). The seller addresses her with a greeting (6), while manipulating the card reader: he quickly turns his head and gaze to her (5–6) at the beginning of the greeting, then promptly gazes back at the reader where he initiates typing the price. In this way, he both manages to establish mutual gaze during the greetings (fig. 5) and to display continuing his current action, progressing the payment.

Moreover, without looking at her anymore, he adds an announcement of his availability (9): in this way, he makes publicly accountable that the closing of the current encounter is imminent, projectable on the basis of paying. Then he continues with the payment.

Greeting sequences are thus skillfully inserted in the current encounter while at the same time displaying a main involvement in it, in sequential environments where a momentaneous disengagement with talk to the current customers is possible and legitimate, designed as expectably short and as not hampering the progressivity of the current course of action.

#### Announcing imminent/future availability

In extract 2, the seller does not only greet but adds an announcement about his imminent availability. With these announcements, the seller addresses future customer waiting by displaying that they monitor their position in the queue, projecting their imminent turn. Announcements recurrently take the form *je viens tout de suite/*“I come right away,” *je suis à vous tout de suite*/“I am yours right away,” or *j’vous oublie pas*/“I don’t forget you.” These announcements of imminent service can be produced at different moments within the current transaction and in response to different conducts of the waiting customers. They display a double orientation of the seller toward the progression of the current encounter (as it can also be overseen by the next customer) and the approach and posture of the waiting customer(s).

While the greetings target new customers approaching, the announcement is addressed to waiting customers and is sensitive to particular sequential environments within the overall structural organization of the service encounter, that is, positions enabling a projection of its imminent closure. Typical environments are when the current customers are paying (extracts 2, 3), when the seller is preparing the wrapped bag of the purchase, or even when he is fetching the requested product.

In the next extract, the announcement is produced as payment is being completed:

**(3**) (**1.09.55—CLI17/CLI19/CLI20)**

**Figure:**
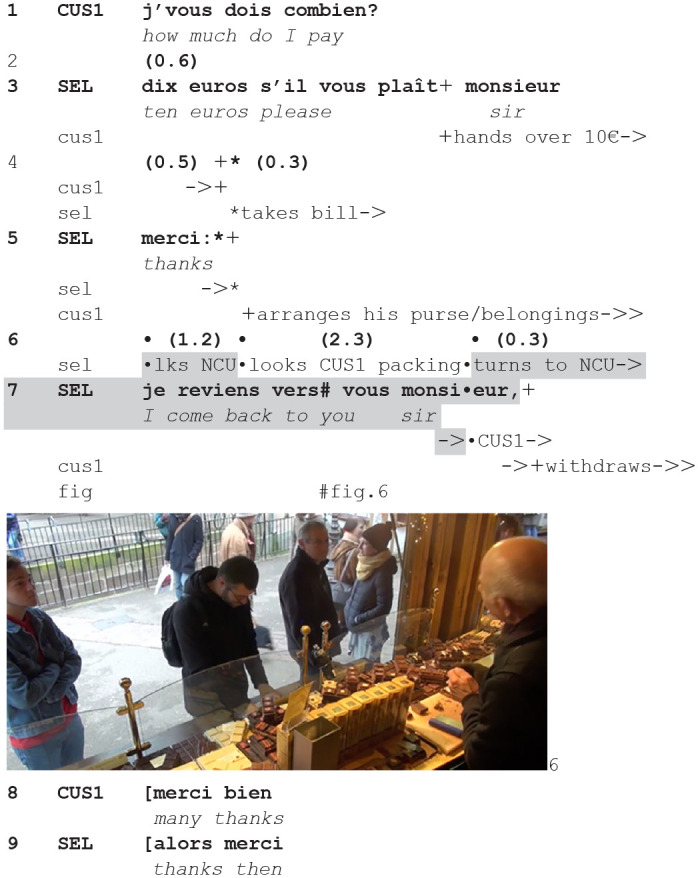


The current customer initiates the payment sequence (1) and upon the announcement of the price (3), gives out the exact change (3–4) to the seller who thanks him (5). At this point emerges a lapse: the current customer is arranging his personal belongings, not looking at the seller anymore. The seller alternates his gaze between the next customer waiting, and the current customer finishing packing, and back (6). This shows a double orientation as the current encounter is not yet closed and the next is not yet opened (cf. Mondada, in press). It is also a slot in which the current customer is not available for progressing action while the next one is co-present waiting. The seller produces the announcement (7, fig. 6) in this sequential position. The announcement makes for the next customer the closing explicitly projectable. Next, indeed, the current customer and seller engage in the final greeting sequence, as the former has already begun to bodily withdraw from the counter.

Another environment in which the imminent closing is projectable is the wrapping of the purchase, as here:

**(4**) (**1.19.10—CLI24.1/2,CLI26.1/2)**

**Figure:**
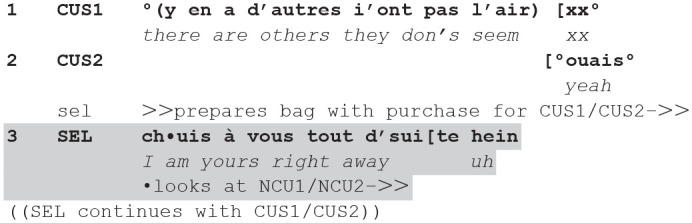


In this case, the seller is wrapping the purchase and the current customers are talking to each other about the displayed products (1–2). The seller exploits this moment in which the current encounter is not progressing with him for producing the announcement (3), which also orients to imminent closing.

While in the previous cases the announcement is uttered when the closing of the current transaction is clearly projectable, in some cases the announcement is produced earlier:

**(5**) (**2.29.00-2.30.07—CLi46.1/2-CLI47.1/2)**

**Figure:**
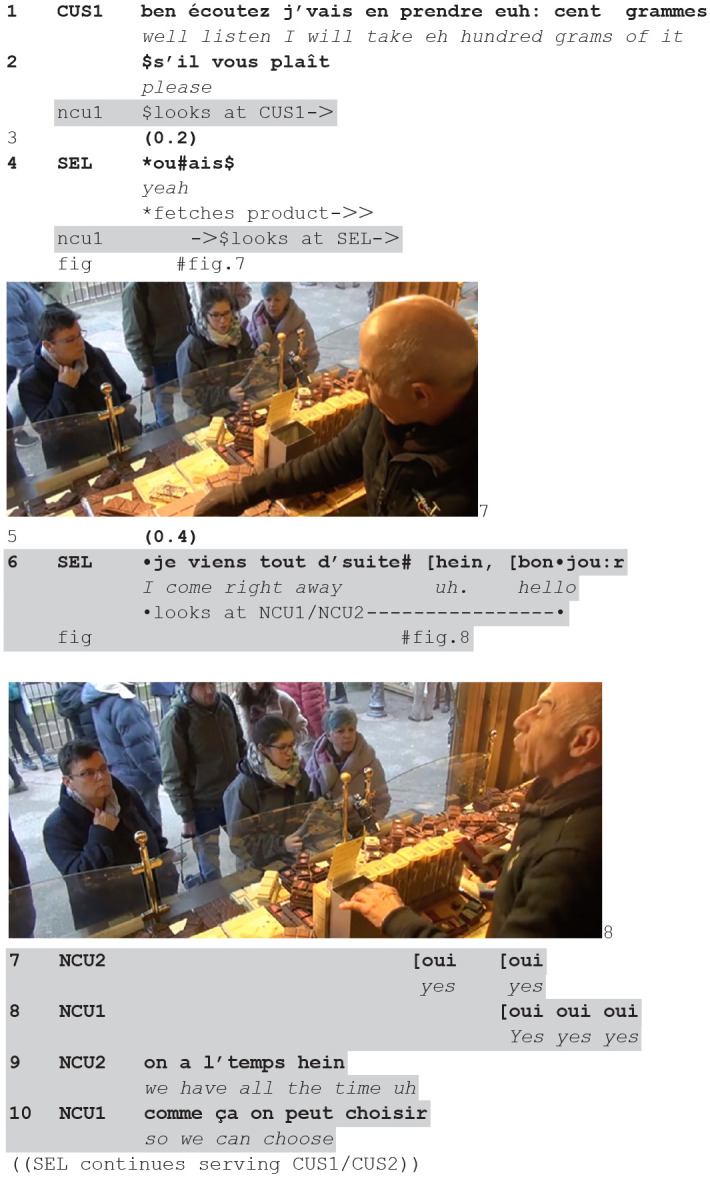


The current customer (CUS1) makes a request (1–2) and the seller grants it both verbally and bodily by fetching the product (4). When she utters the request, the new customer, beside her, looks at her (fig. 7), and then at the seller. This series of gaze is seen by the seller, who responds to them, gazing back, with an announcement of an imminent service (6, fig. 8). The fact that he utters first the announcement and then the greeting, as well as the particle *hein?* confirms that his turn is a response to the new customer’s gaze—perhaps interpreted as irritated or annoyed. In return, the two next customers respond by dismissing urgency. So, they respond to the seller’s turn and to his orientation to possible problems of time, providing for accounts. The seller then continues to serve the current customers.

The seller exploits—as in the previous cases—a moment in the service that is located after the last sequence has been completed, as there is not any talk going on, and any conditional relevance projecting an expected action from him. Engaged in fetching the requested product, he can turn to the next customers while bending over the products, and address them while at the same time continuing the action that progresses the current encounter. Moreover, his announcement orients to the observable conduct of the queuing customers, possibly ascribing an orientation to time, waiting, and some possible urgency. Thus, the initiation of a sequence addressing other customers than the current ones, and embedded in the current transaction is sensitive both a) to the ongoing transaction with the current customer(s), favoring sequential positions within it in which the intersected actions do not hamper its continuity and progressivity, and b) to the conduct of the waiting customer(s), considering how they wait, the time they have been waiting, and how projectable is their own access to the service, displaying them they are taken care of without yet selecting them as currently served.

### Managing service

In the previous section, I focused on short, circumscribed, embedded sequences timely inserted in the ongoing encounter without obstructing its progressivity, and without engaging with the new customers, rather announcing and projecting their imminent/future turn-at-service. By contrast, other sequences can do more than that, that is, engage in some service/selling with the new customer(s) even if it is not yet their turn. While the insertion of greeting or announcement sequences is not oriented normatively by the current customer, the initiation of some service is less unproblematic, and might be oriented to as deviant. This section addresses the issue of how consequential the intersected actions relative to a new transaction can be relatively to the progressivity and management of the ongoing one.

#### Offering to taste a product’s sample

In a food stall, offers to taste a product’s sample are a recurrent commercial strategy, stimulating the interest for the product or supporting an ongoing choice between various options (Mondada, 2023b). In the context studied in this paper, offering a taste is also an action that can address other customers than the current ones, and that can be embedded in the ongoing transaction.

The offer to taste addressed to new customers is often related to a similar offer to current customers performed just before. Here is an instance, in which the seller is offering a taste to the current customers (CUS1, CUS29, after they already bought some chocolate.

**(6**) (**1.17.30-CLI24.1/2-CLI26.1/2)**

**Figure:**
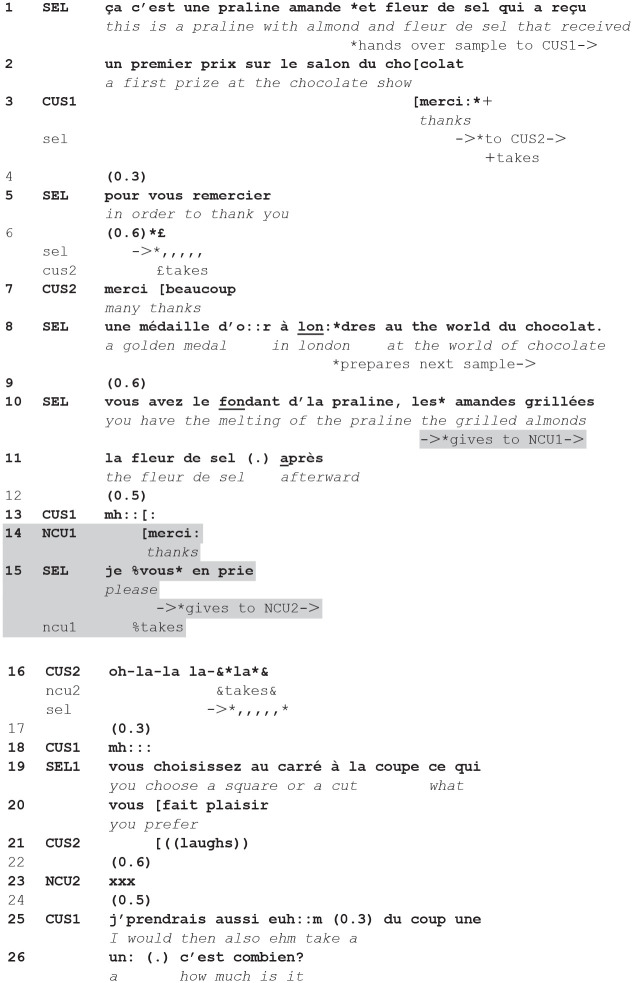


The seller hands over a sample to the two customers, while he describes the product (1–2). As they thank him (3, 7), he formulates the offering as thanking them for their purchase (5). He then continues the description (8–11), while preparing and then handing over a sample to the next customers (10, 15). In this way, the new customers taste the same as the current ones; they can hear the same description, and overhear the customers positively assessing the sample (13, 16, 18). The description is completed with information about possible formats in which to buy the product (19–20), addressed to the current customers but even more to the next ones. So, the fact of offering a sample to taste occasions the possibility to share with the new comers much more than just the material sample; rather, this offer occasions extended talk about the products, which is particularly relevant for the new customers (since the previous have already made their purchase). The fact that these explanations are geared toward selling is observable in the final customers’ request (25–26).

While in the previous extract the offer was done silently, just handing over the sample on a knife used as a board, in the next excerpt the offer to taste is introduced verbally (10), ascribing the willingness to taste to the new customer:

**(7**) (**2.35.36-cli47.1/2-cli48 offer to taste while current tastes)**

**Figure:**
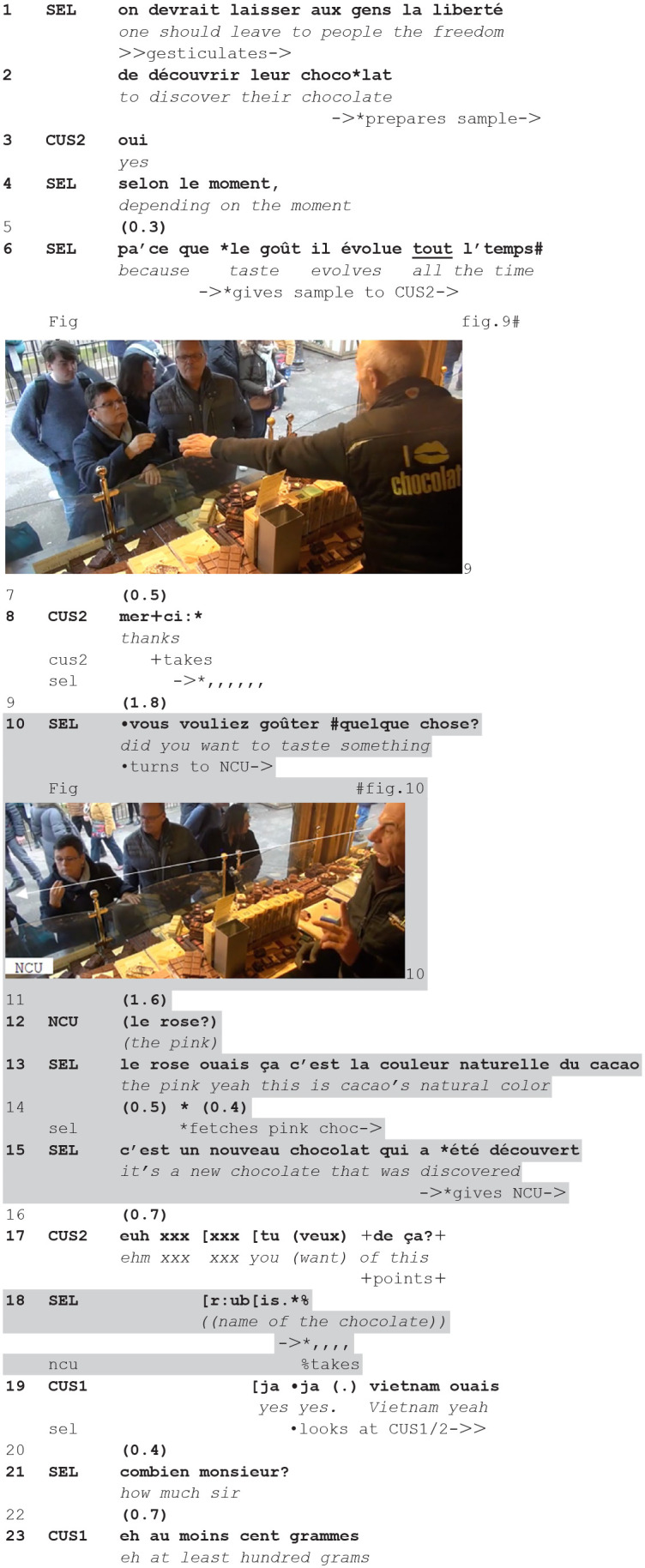


The seller is theorizing about taste (1–6), and offers a sample to the current customer (CUS2) who had previously asked a question about it (fig. 9). The customer takes it and thanks (8). A lapse emerges during which the customer is silently tasting.

At that point, the seller turns to the new customer (only partially visible on the left of the video frame), and offers her to taste something (10, fig. 10). She accepts and proposes a chocolate she identifies on the basis of its color (12), occasioning a description/explanation about that chocolate (a new creation) (13–15) and its name (18), while the seller fetches it and gives it to the new customer.

While the seller gives out the sample and its name, the current customer (CUS2) engages in a sequence with her partner (CUS1), about a chocolate they seem to be interested in. The seller overhears the exchange and immediately turns toward them, ascribing to them a willingness to buy something, and asking how much they want (21), promptly responded to by CUS1 (23).

So, the seller exploits a lapse in the current interaction to initiate a new sequence, with new participants, which consists not just in an offer and its acceptance but also in a more elaborated expansion, being completed by the fetching of the targeted sample. At the same time, this gives the current customers the opportunity to exchange about what they want next. Interestingly the seller demonstrates to be monitoring them, and promptly turns back to them, repristinating the continuity of their transaction.

These two instances of embedded offer to taste show how it can be minimally impinging on the ongoing transaction (extract 6) or it can be more extended (extract 7). In both cases, however, the seller displays an orientation to the progressivity and the priority of the ongoing (current) course of action: when the intersected sequence develops in a more extended way, he manages to embed it in a skillful way in the current encounter, so to minimally hamper its progressivity.

#### Border and deviant cases: Limitations to what can be intersected

Offers to taste are an exemplary case of sequences of action that can be intersected in an ongoing activity without suspending it and without the participants orienting to it as normatively violating some rights or primacies. This shows that the current encounter can be preserved despite integrating various types of sequences, such as greetings, announcements and offers. This raises questions concerning the limits of what can be intersected in that way. Are there types of sequences that can be inserted more legitimately by contrast to others that would normatively appear as more problematic? In order to explore these issues, we focus in this section on cases in which some sequences are treated as less acceptable than others.

Roughly, the next two fragments show that whereas inserted offers are often not a problem, the expansions they can generate—such as questions and requests—might cause some trouble. In particular, questions by new customers are generally accepted, but their requests are generally rejected. This shows a distinctive orientation of the participants to actions having different forms of consequentiality and representing different forms of engagement, relatively to the current “main” course of action.

Instances of these actions are observable in the next extract, in which the seller, while serving the current customer (CUS), silently gives a sample to taste to a new customer (1), who then initiates a question-answer sequence about it (4), and then moves to a request (15). While the question is answered, the request is rebutted (delayed).

**(8**) (**48.24-49.52/B—CLI9-CLI10.1/2)**

**Figure:**
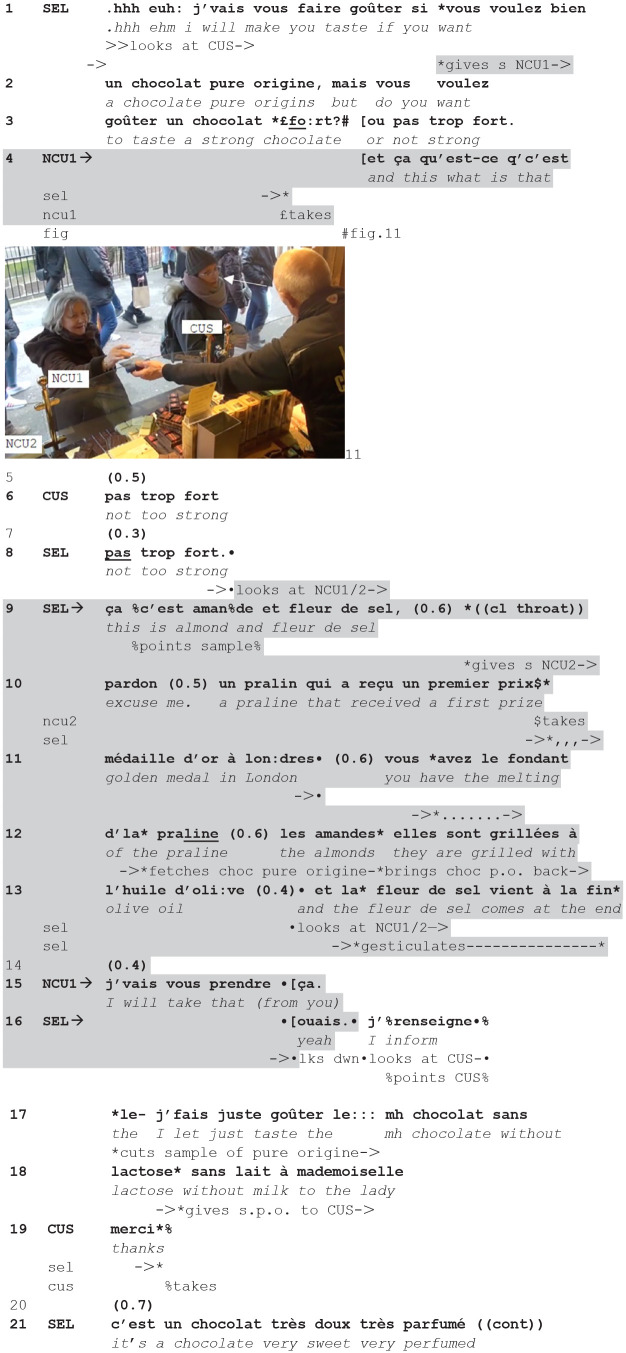


The seller is serving the current customer who requested a chocolate without milk. He proposes a “chocolate pure origins” (2) immediately offering two possible alternatives to choose from (“strong” vs “not strong” 3). The customer picks the latter option (6) and the seller confirms (8).

During this sequence, the seller also gives two new customers another chocolate to taste, with almond and *fleur de sel*: he offers a sample silently, just by handing it over to NCU2 (1), and then to NCU1 (9). As visible in fig. 11, the seller engages at that moment in a body torqued posture (Schegloff, 1998), bending toward NCU1 with his extended left hand offering the taste, while looking at CUS. This body torque reveals his way of engaging in a form of multi-activity, and in two distinct participation frameworks.

NCU1 takes the sample (4) and immediately asks “and this what is that” (4). The seller responds (9), but only after having completed the previous sequence with the current customer concerning her choice. So, sequentially, he displays the priority he gives to the ongoing transaction with the current customer. However, as soon as he has confirmed her choice, he turns toward the new customers: He aligns with the question by beginning his answer with the same turn-initial pronoun *ça*/“this” (9, cf. 4), and expands it with a description of the chocolate (9–13). During this extended description, as soon as he has given out two samples to the two new customers, to whom the description is addressed, he turns toward the counter and moves along it to fetch the chocolate for the current customer (11–12), which he brings back to the counter (12–13) before cutting it (17–18). Here too, the seller engages in multi-activity: he uses talk to describe the sample tasted by the new customers, while using his body to fetch the sample for the current customer.

At completion of the description addressed to the new customers, he looks at them, and gesticulates to them (13). NCU1 responds to the description with a request to buy the product (15). At this point, the seller briefly aligns with the request with *ouais*/“yeah” (16) but lowers his gaze and immediately continues—while shifting gaze to the current customer—delaying the granting of the new customer’s request in favor to an action addressing the current customer, giving her a taste (self-repaired 16–18). This action is both performed (he cuts the sample of pure origins) and formulated (17–18). The formulation refers to the current customer in the third person, thus addressing the new customers, as an account.

In this extract, an offer to taste to new customers, inserted into the current service, expands into a question/answer about the tasted sample first, and second into a request to buy the product. The seller manages and aligns to the former in engaging in multiactivity, but delays the latter, by explicitly prioritizing the current encounter. In this way, he makes clear how he manages the current versus the new encounter: he prioritizes the current until the completion of a verbal sequence (8), then manages both thanks to a form of multiactivity in which his talk addresses the new versus his body addresses the current course of action, and finally prioritizes the current one again, delaying the new one. Moreover, the seller’s management of the question and the request generated by his offer, manifest a differentiated orientation toward the rights and obligations related to these actions: while the question generates an obligation to answer that can be managed by still serving the current customer, the request implies an obligation to grant it that de facto conflicts with the ongoing (current) transaction and means suspending it. The way the seller responds reveals his hierarchization of the sequences that can be inserted in the ongoing course of action: while question/answer is treated as acceptable, request/granting the request is treated as problematic—jeopardizing the priority of the current encounter. In this sense, the extract reveals how issues of priority, legitimacy, and entitlement emerge not just in the management of multiactivity, but in the management of hierarchically ordered courses of action.

These orientations are recurrent in the data, as the next and final fragment reveals.

**(9**) (**1.21.20-CLI26.1/2-CLI27)**

**Figure:**
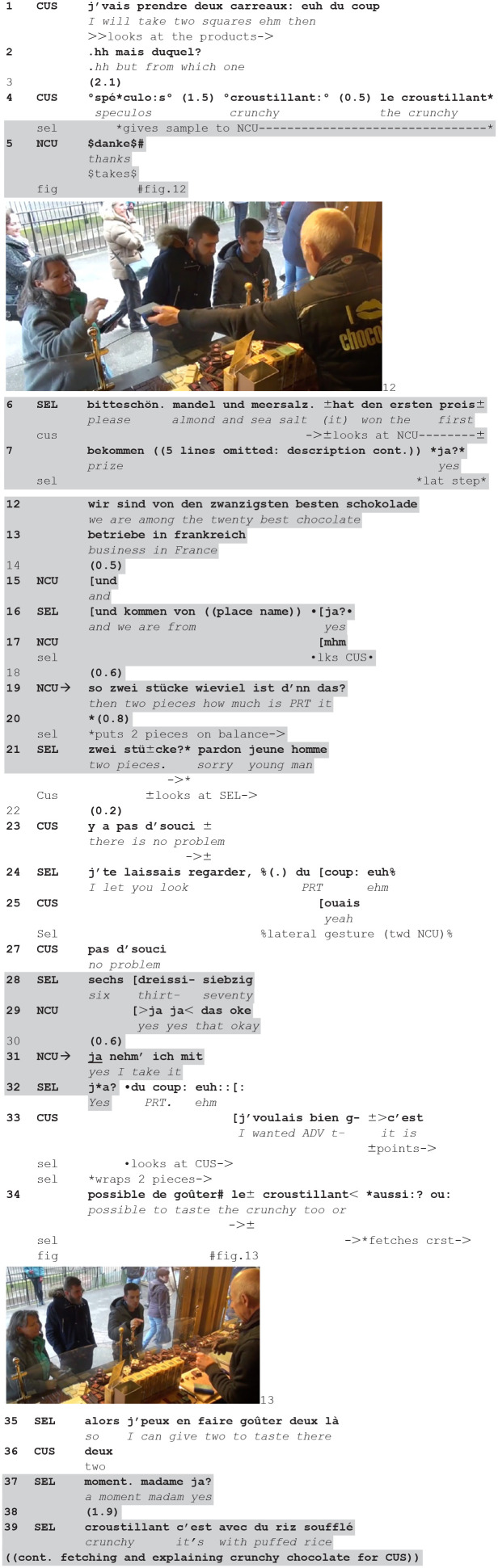


The seller is serving the current customer (accompanied by a friend), who is deciding to buy two squares (1) but hesitates between various types of chocolate to choose from (2 to 4). The seller exploits this moment of choice to give a sample of another chocolate to the next customer (4) who thanks him in German (5). The current customer is still looking at the stall (fig. 12) and the seller engages in a description—also in German—of the sample (6–16). During this description, the seller ends twice a TCU with the particle *ja?*/“yes” (6, 16), the first with a lateral step (moving from NCU to CUS), the second looking up at CUS. At these possible completion points, he bodily orients back to the current customer. But in the first case, he also continues his information delivery, and in the second, it is the new customer who exploits the possible relevant transition point to ask a question. So, despite an orientation projecting a possible come back to the current customer, the seller ends up continuing the interaction with the new customer.

So after having tasted a sample, the new customer asks a question about the price (19). In response the seller weights the referred to quantity (20). He repeats the quantity (21), but at that point orients to the current customer—who is looking at him— and apologizes (21). The position at which the seller switches back to the current customer, in the middle of a sequence with the other customer, and the fact that he does so with a specific action—apologizing—displays that this sequential position and this emerging sequence of actions could be seen as problematic. Even if the customer gives a go-ahead (23), the seller continues with a retrospective account which topicalizes the conditions at which he engaged in inserting an exchange with the next customer—in order to let the current customer look and choose (24). The account is organized in two parts, the second (*du coup: euh*/“suddenly/consequently” + lateral gesture indicating the new customer, 24) although incomplete describes the (unintended) shift to the new customer. Again the customer dismisses the problem (27, cf. 23).

This is treated by the seller as a go-ahead for pursuing his interaction with the new customer: he gives the price that she asked for (28, responding to 19). So, the sequence question/answer is completed after the second pair part being delayed by an orientation to another ongoing course of action and the legitimacy of prioritizing it.

This occasions another action by the new customer, retrospectively showing that the question about the price was a pre-sequence, projecting a request (31). The seller repeats interrogatively the particle *ja?*/”yes” that she was also using (32, cf. 31) (which is also the one he was using before, while orienting back to the initial customer, cf. 6, 16) and indeed turns again to him, switching into French (32). The seller uses again the particle *du coup*/”suddenly/consequently” (32, cf. 24). The interpretation of the particle is multiple, and can be tilted toward temporality (“suddenly”) but also toward consequentiality (“consequently,” “therefore,” “so”). While the seller seems to use it to ask for permission to continue to serve the new customer, the recipient does not treat it that way, but rather as an opportunity to progress his own transaction. Thus, the customer self-selects (33) requesting to taste a sample of *croustillant*. His request is self-repaired: it begins with a modal verb in the past, *j’voulais bien*/“I was well willing” 33 which seems to refer to his long maturation of his request, as well as to the time he waited before to utter it, and then repairs it into *c’est possible de goûter/*“it’s possible to taste” (33–34, fig. 13), with an interrogative prosody and a declarative syntax—displaying entitlement to the request. Moreover, the request is uttered with an accelerated pace, orienting to the possible competition with the new customer. In response, the seller, who was already wrapping the products requested by the new customer, abandons this action, leaving the bag and the product on the counter, to fetch the sample to taste. The manner in which this action of packing, which was almost complete, is abandoned, can be a way for the seller to display to the customers (both the current and the new one) which course of action has priority and legitimacy. As a matter of fact, the seller does not only fetch the requested sample, but turns to the new customer explicitly referring to the suspension (37) of the exchange with her.

So, this episode confirms that sellers might find themselves in a challenging situation, created by the offer to taste initiated by them but also by other actions expanding it, in which he is confronted to the dilemma of either responding to the actions of a new customer, thus violating the integrity and the order of the service to the current customer, or blocking and delaying them to respect the order of the service and prioritize the initial customer.

## Conclusion

The analyses have unpacked the sequential organization of how interactions can be intersected in an ongoing encounter. Focusing on actions initiated by the seller in service encounters-in-a-series, addressing other customers than the current ones, the analyses have considered how they are accountably positioned and built as legitimate, as respecting the priority, rights and autonomy of the current customer, while maximizing the local participation of other customers waiting and queuing. The analyses have revealed a distinctive normative orientation toward different actions that can be inserted in the current encounter: not all the sequences of actions initiated by the seller, or subsequently by the customer he has initially addressed, are treated as equally legitimate. For instance, greeting sequences (to possible new customers approaching the counter) as well as announcements of imminent availability (to next customers waiting to be served) are generally unproblematically inserted in the current transaction. In particular the latter exhibit an orientation to the order of the service, categorizing de facto the recipient as “next” in the queue. However, the position in which they are initiated by the seller reveals a delicate attention to the sequential expectancies related to the current encounter: the seller skillfully positions these actions in sequential environments in which there is an accountable lapse of some sorts—the current sequence has been brought to completion, or the current customer is taking time to produce an expected action (like deciding which product to select), or the seller is performing a manual action taking some time (like fetching or wrapping a product, or preparing payment)—that enables a double involvement without hampering the progression of the current encounter (§4.1). More problematic sequences of actions have been identified (§4.2), which are related not only to expansions of sequences initiated by the seller, taking more time and engaging further the involvement with the new customer, but also to actions initiated by the customer which imply a transformation of rights and obligations. So, whereas questions asked by another-than-the current customer are generally answered by the seller, even at length, requests following questions about the product are generally not granted by him, but delayed to another moment, treating the new customer as “next” in the queue, who will be become later the “current” customer. This differentiation of actions addresses issues of legitimacy, rights and priorities. An intersected request modifies the order of service, claiming rights to be served that suspend the rights of the current customer, thus problematizing the integrity, priority and progressivity of the current encounter. So, questions versus requests are normatively oriented to in very different ways: questions are actions treated as not (yet) constituting a transaction, whereas requests are treated as implicating specific economic (and moral) rights and obligations, as well as other consequential actions (like paying). Requests are treated as modifying the order of the service by jumping the queue. While the seller has specific responsibilities and authority in initiating and responding to actions, and does some policing work when new customers initiate actions entering in competition with the rights of the current customers, the navigation of these diverse normative implications is ultimately a collective matter of all the participants.

So, intersecting sequences of action in an ongoing encounter reveal the members’ orientations toward the issue of the overall structure of the encounter and the order of encounters-in-a-series: the integrity of the encounter does not exclude any intersecting action by other participants, and therefore does not exclude some form of porosity. However, the participants orient to the integrity of the encounter for distinguishing between unproblematic versus illegitimate intersecting actions and sequences of actions. This shows how the integrity and autonomy of social interaction is indeed a members’ concern, and is locally managed by members. This shows how the normative orientations of the participants intervene in crucial ways in the collective management of the rights and obligations not only characteristic of types of participants but also attached to particular sequences of actions, and sequencing orders, thus recognized as fundamental features of sequence organization.
